# The contribution of child, family and health service factors to respiratory syncytial virus (RSV) hospital admissions in the first 3 years of life: birth cohort study in Scotland, 2009 to 2015

**DOI:** 10.2807/1560-7917.ES.2019.24.1.1800046

**Published:** 2019-01-03

**Authors:** Pia Hardelid, Maximiliane Verfuerden, Jim McMenamin, Rosalind L Smyth, Ruth Gilbert

**Affiliations:** 1 UCL Great Ormond Street Institute of Child Health, London, United Kingdom; 2 Health Protection Scotland, Glasgow, United Kingdom

**Keywords:** respiratory syncytial virus, respiratory viruses, RSV, vaccines and immunisation, infant, child, preschool

## Abstract

**Introduction:**

Several vaccines for respiratory syncytial virus (RSV) are under development. Designing an effective vaccination programme for RSV requires information about the relative contribution of risk factors for severe RSV symptoms.

**Aim:**

To inform preventive strategies in Europe by quantifying the contribution of key child, family and health service risk factors to the burden of RSV hospital admissions in young children.

**Methods:**

We constructed a birth cohort study of all singleton children born in Scotland between October 2009 and September 2012 using linkage between birth registration, maternity, vaccination and hospital admission records, with follow-up until the age of 3 years. RSV-confirmed hospital admissions were defined using linkage to national laboratory surveillance data. We estimated hospital admission rates per 1,000 child years and length of stay according to each risk factor. Cox proportional hazard regression models were used to estimate adjusted hazard ratios.

**Results:**

There were 5,185 RSV admissions among the 169,726 children in the cohort: 48.6% of admissions occurred before the age of 6 months, and 29.6% after the age of 1 year. Children born prematurely, small for gestational age, between July and December, with chronic conditions, older siblings, mothers < 30 years old or delayed infant vaccination had a significantly increased risk of admission. Minimising the risk posed by older siblings could reduce RSV admissions by up to 34%.

**Conclusion:**

Future RSV vaccination programmes must protect children throughout early childhood. Vaccination and/or interventions to reduce transmission by older siblings could substantially reduce RSV hospital admissions.

## Introduction

Respiratory syncytial virus (RSV) is a frequent cause of respiratory infection and the main cause of bronchiolitis [1], the most common reason for hospital admission in infants in the United Kingdom (UK) [2] and many other developed countries. RSV also leads to over 450,000 primary care consultations in children in the UK every year [3]. In childhood, symptoms of RSV infection are more severe at the youngest ages [4]. Further, RSV infection in young infants has been linked to wheezing and asthma in later childhood [5,6].

There is a lack of cost-effective preventive strategies for RSV infection. Palivizumab, a humanised monoclonal RSV antibody, has been shown to reduce hospital admissions in children born preterm or with congenital heart disease [7], but is costly [8]. It is therefore only recommended in children at high risk of serious complications, who have chronic heart or lung conditions or immunodeficiency [9]. However, over 80% of infants who are admitted to hospital with bronchiolitis in the UK are born at term and otherwise healthy [10].

RSV vaccination is likely to be the most effective preventive strategy, and several vaccine candidates are under development [11]. Options include targeting pregnant women, infants or older children. Vaccine strategies need to account for the short period of immunity following natural infection [12] and the young age of children with the most serious complications of RSV. Older siblings are an important source of infant infections in low-income country settings [13], however, there have been few studies of the contribution of older siblings to the burden of RSV hospital admissions in high-income countries [14]. The design of an effective RSV vaccination programme needs information on risk factors in order to effectively prevent the maximum number of cases and severe complications. A systematic review identified few large-scale studies of multiple risk factors for confirmed RSV infection in economically developed countries [15]. Linkage of health administrative databases, including hospital admission data, offer a whole-population cohort design for investigating risk factors for RSV-related hospital admission [10,16-18]. These studies have highlighted the importance of clinical risk factors for RSV-related admissions in infancy, such as prematurity and chronic heart and lung disease. However, estimation of risk related to family structure is difficult as such data are limited in electronic hospital databases and diagnostic coding is unreliable for specific infections such as RSV [19,20].

In this study we examine the relative contribution of child, family and healthcare risk factors for RSV-associated hospital admission in the first 3 years of life to inform vaccination and other preventive strategies.

## Materials and methods

### Data sources, study population and period

We developed a birth cohort study of all singleton children born in Scotland to Scottish resident mothers between October 2009 and September 2012, using linkage between the following national administrative health databases: birth and death registration records, maternity records (the Scottish Morbidity Record; SMR-02), Scottish Birth Records (which records neonatal diagnoses), hospital admissions (SMR-01) and the infant vaccination registry (Scottish Immunisation Recall System, SIRS). RSV-confirmed admissions were identified via linkage to the Electronic Communication of Surveillance in Scotland (ECOSS) database, a public health surveillance database held by Health Protection Scotland (HPS), the national infection control agency. We excluded children with a birth weight less than 500 g and children born at less than 24 weeks to ensure exclusion of stillbirths. Deterministic linkage between databases was carried out using the Community Health Index (CHI) number, a unique individual identifier used across the Scottish National Health Service (NHS Scotland) from birth [21]. The completeness of CHI number in the datasets that were linked by the electronic Data Research and Innovation Service (eDRIS) for this study was very high, including 99.6% of records in birth registration data, 99.7% in Scottish Birth Records, and 99.8% in SMR-01 (Carole Morris, eDRIS, personal communication, June 2018). Linkage was carried out by eDRIS, and only de-identified data was made available to the research team.

Children were followed from birth until their third birthday, their date of death or the date of moving out of Scotland (as recorded on the national CHI database), whichever occurred first. Outcomes were measured in the period 1 October 2009 to 30 September 2015 to ensure all children were followed up for 3 years from birth (unless they died or emigrated before this).

### Outcome

An RSV-confirmed hospital admission (hereafter referred to as RSV admission for brevity) was defined through linkage between SMR-01, the national hospital admission database, and ECOSS. ECOSS contains reports of positive test results reported by participating microbiology laboratories serving NHS hospitals in Scotland to HPS. Since a child could have multiple positive tests during the course of one illness, HPS link positive ECOSS reports of the same pathogen in the same patient into infection episodes based on the specimen collection dates. We defined an RSV admission as any non-injury-related emergency hospital admission with a linked positive RSV ECOSS episode with a specimen date up to 7 days before or after the hospital admission date. Injury-related hospital admissions were defined as admissions where the primary diagnosis was an injury (International Classification of Diseases version 10 (ICD-10) codes S00-T79 [22]). If a child had more than one hospital admission within the 14-day window of an RSV-positive ECOSS episode, we selected the admission with the admission date nearest to the specimen collection date as the RSV-associated admission.

### Risk factors

#### Child-level risk factors

Gestational age was classified into a four-category variable using an established classification [23]: extreme, severe and moderate preterm (< 34 weeks), near-term (34–36 weeks), term (37–40 weeks) and post-term (≥ 41 weeks). Birth weight for gestational age categories were derived using birthweight centiles for Scottish children [24]. Small and large for gestational age was defined as having a birthweight less or greater than the 10th percentile for the particular gestational age, respectively. Children who were neither large nor small for gestational age were categorised as normal for gestational age. We used a previously developed list of ICD-10 codes to identify children with chronic conditions, including chronic heart, lung and neurological conditions, who are at increased risk of RSV-related complications [25]. We searched for the relevant codes in Scottish Birth Records and during longitudinal hospital records up to the age of 6 months. Annual quarter of birth was coded into a four-category variable (January–March, April–June, July–September, October–December). Apgar score at 5 minutes was coded as a binary variable (0–7 and ≥ 8). Prolonged postnatal stay after delivery was used as a further indicator of early-life neurological or respiratory problems with potential long-term sequelae, and was coded into a binary variable: ≤ 14 days or > 14 days.

#### Family-level risk factors

Number of siblings at birth was derived using the parity variable in SMR-02 maternity records, and coded into a three-category variable: no siblings, one sibling or two or more siblings. Maternal smoking during pregnancy was coded as a binary variable (yes/no). Socioeconomic status was measured using maternal age and area-level deprivation. Maternal age was coded into a four-category variable (< 20, 20–29, 30–39 and ≥ 40 years). The Scottish Index of Multiple Deprivation (SIMD) is an area-level indicator of deprivation derived from several indicators (mainly from the UK Census) [26]. SIMD scores are calculated at small-area level, where each area includes 500 to 1,000 persons. SIMD scores were grouped into quintiles and linked to the child via the maternal postcode at delivery.

#### Health service-related risk factors

We examined vaccination delay as an indicator of access to preventive health services. Children in the birth cohort should have received three doses of pentavalent (diphtheria/tetanus/pertussis/polio/*Haemophilus influenzae* type B) vaccine and two doses of pneumococcal conjugate vaccine by 4 months of age. To examine the association between vaccination history and risk of RSV admission we defined a binary variable indicating delayed infant vaccination if a child had not received all required doses by the age of 6 months.

### Statistical analyses

All statistical analyses were carried out using Stata version 13 (StataCorp LP, College Station, TX, USA). We determined the number of RSV admissions according to month of age in the first 3 years of life, and estimated RSV admission rates per 1,000 child years by year of age and each risk factor. We estimated the median and interquartile range (IQR) of the length of stay of the RSV admissions according to each risk factor, and the proportion of total bed days (that is, the total number of days in hospital) during RSV admissions by each of the risk factors. Kruskal-Wallis tests were used to compare length of stay distributions. To calculate bed days, children with a length of stay of 0 days were allocated 0.5 bed days.

Only the first RSV admission for each child in was included in the statistical models, and children who were admitted were censored at their admission date. We used Cox proportional hazards regression models to estimate adjusted hazard ratios according to each individual risk factor, adjusted for all others. The proportional hazards assumption was checked using cumulative hazards plots. We included all risk factors in the model a priori. A separate model was fitted to evaluate the effect of delayed infant vaccination (since we defined this variable at age 6 months), with follow-up started at age 6 months, rather than at birth. Since there was a non-negligible proportion of missing data on key risk factors, we used multiple imputation with 15 imputations to estimate model parameters. A Wald test p value < 0.05 was used to determine whether a particular model parameter was significantly associated with the outcome. Complete case analyses were carried out as sensitivity analyses. Population attributable fractions were calculated for all risk factors which were significantly associated with the outcome using the *punafcc* command in Stata [27], from the complete case models. For the population attributable fractions, we assumed that the observed associations between each risk factor and RSV admission risk were causal.

#### Ethical approval

The study was approved by the Public Benefit and Privacy Panel for Health and Social Care, reference number 1516–0405.

## Results

**Figure 1:**
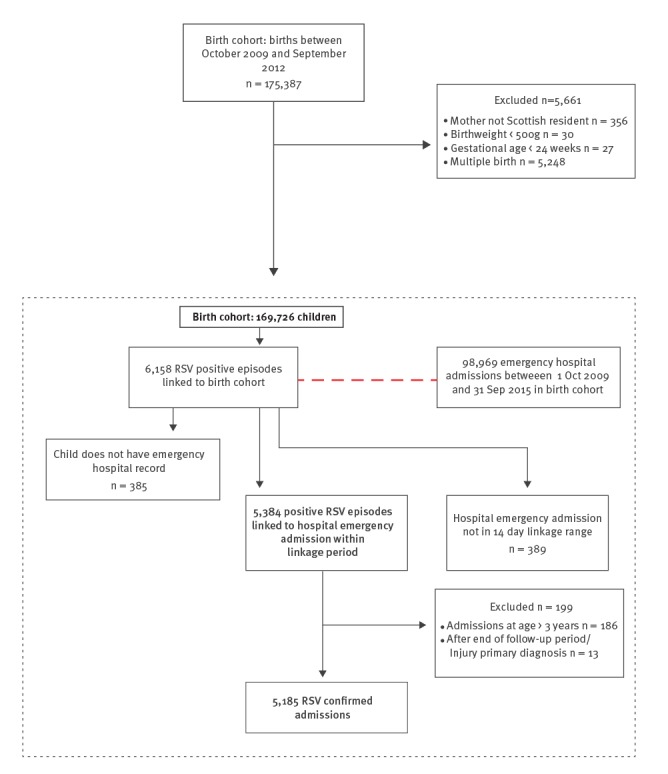
Flow chart of derivation of the final study cohort, respiratory syncytial virus hospital admissions birth cohort study, Scotland, 2009–2015 (n = 169,726)

The cohort included 169,726 children, who were followed for an average of 2.95 years. The characteristics of children in the cohort are shown in Table S1, and the linkage outcomes in Figure 1. There were 6,158 RSV-positive ECOSS episodes linked to the birth cohort, of which 5,384 (87.4%) were linked to a hospital admission within 14 days of the sample date.

RSV: respiratory syncytial virus.

**Figure 2:**
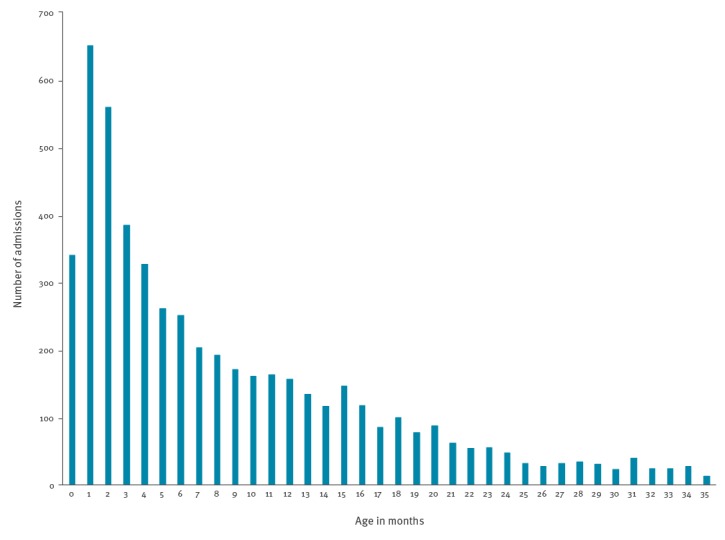
Distribution of respiratory syncytial virus hospital admissions by month of age in children less than 3 years old, birth cohort study, Scotland, 2009–2015 (n = 5,185)

There were 5,185 RSV admissions in 5,033 children: 4,884 children had one RSV admission (97.0%), and 149 (3.0%) had two or more during the first 3 years of life. Of the 5,185 RSV admissions, 19.1% (n = 989) occurred before the age of 2 months (Figure 2), and nearly half (48.5%) before 6 months (n = 2,517). A further 29.6% (n = 1,532) of RSV admissions during the first 3 years of life occurred after the age of 1 year.

Only 19.4% (n = 965) of the 4,966 RSV admissions in children with a known gestational age were among children who were either premature or with an underlying chronic condition. However, the median length of stay was significantly longer among premature babies and children with chronic conditions (Table 1). The overall median length of stay was 2 days but children born at less than 34 weeks or with chronic conditions had a median length of stay of 3 days. Children with known chronic conditions accounted for 11.1% of admissions but 20.4% of bed days.

**Table 1:** Distribution of respiratory syncytial virus hospital admissions, and median and interquartile range of length of stay in hospital according to risk factor, birth cohort study, Scotland, 2009–2015 (n = 5,185 admissions)

Risk factor	Number of RSV hospital admissions	% of RSV hospital admissions	Median length of stay (IQR) in days	Kruskal-wallis test with ties p-value for difference in median length of stay	Number of bed days^a^	% of bed days
**Gestational age**						
< 34 weeks	268	5.2	3 (1–6.5)	0.0001	1,640.5	10.7
34–36 weeks	335	6.5	3 (1–5)		1,470.5	9.6
37–40 weeks	3,431	66.2	2 (1–4)		9,306	60.6
≥ 41 weeks	932	18.0	2 (1–4)		2,212	14.4
Missing	219	4.2	2 (1–4)		734.5	4.8
**Sex**						
Boys	2,876	55.5	2 (1–4)	0.0628	8,580.5	55.8
Girls	2,309	44.5	2 (1–4)		6,783	44.2
**Chronic condition recorded before 6 months**						
No	4,611	88.9	2 (1–4)	0.0001	12,235	79.6
Yes	574	11.1	3 (1–6)		3,128.5	20.4
**Postnatal stay in hospital**						
≤ 14 days	4,833	93.2	2 (1–4)	0.0001	13,553	88.2
> 14 days	215	4.2	4 (1–7)		1,458	9.5
Missing	137	2.6	2 (0–3)		352.5	2.3
**Season of birth**						
January–March	990	19.1	2 (1–4)	0.0001	3,192	20.8
April–June	977	18.8	1 (0–3)		2,394	15.6
July–September	1,453	28.0	2 (1–3)		3,934	25.6
October–December	1,765	34.0	2 (1–4)		5,843.5	38.0
**Apgar score at 5 min**						
0–7	195	3.8	2 (1–5)	0.0002	995.5	6.5
8–10	4,766	91.9	2 (1–4)		13,596.5	88.5
Missing	224	4.3	2 (1–4)		771.5	5.0
**Birthweight by gestational age**						
Small for gestational age	486	9.4	2 (1–4)	0.7877	1,665.5	10.8
Right for gestational age	3,957	76.3	2 (1–4)		11,501	74.9
Big for gestational age	484	9.3	2 (1–4)		1,377.5	9.0
Missing	258	5.0	2 (1–4)		819.5	5.3
**Number of siblings (parity)**						
None	1,547	29.8	2 (0–3)	0.0001	4200	27.3
1	1,995	38.5	2 (1–4)		5,703.5	37.1
≥ 2	1,435	27.7	2 (1–4)		4,900	31.9
Missing	208	4.0	1 (0–3)		560	3.6
**Maternal smoking during pregnancy**						
No	3,471	66.9	2 (1–4)	0.0021	9862	64.2
Yes	1,246	24.0	2 (1–4)		4,201.5	27.3
Missing	468	9.0	2 (0–3)		1,300	8.5
**Maternal age**						
< 20 years	313	6.0	2 (1–4)	0.1589	981.5	6.4
20–29 years	2,478	47.8	2 (1–4)		6,995.5	45.5
30–39 years	2,076	40.0	2 (1–4)		6,280.5	40.9
≥ 40 years	150	2.9	2 (1–5)		631.5	4.1
Missing	168	3.2	2 (0–3.5)		474.5	3.1
**SIMD quintile**						
1 Most deprived	1,485	28.6	2 (1–4)	0.3251	4,820.5	31.4
2	1,117	21.5	2 (1–4)		3,416	22.2
3	945	18.2	2 (1–4)		2,679.5	17.4
4	888	17.1	2 (1–4)		2,501	16.3
5 Least deprived or missing^b^	750	14.5	2 (1–5)		1,946.5	12.7
**Incomplete infant vaccination at 6 months^c^**						
No	2,125	80.3	1 (0–3)	0.005	5,053.5	77.4
Yes or missing^b^	520	19.7	2 (0–4)		1,475	22.6

IQR: interquartile range; RSV: respiratory syncytial virus; SIMD: Scottish Index of Multiple Deprivation.

^a^ To calculate bed days, children with a length of stay of 0 days were allocated 0.5 bed days.

^b^ Fewer than five children had a missing value.

^c^ Including RSV admissions occurring after 6 months of age only. Total number of admissions: 2,645. Total bed days: 6,528.5.

The 5,185 admissions were in 169,726 children, with total of 15,363.5 bed days.

The overall RSV admission rate in the first, second and third year of life was 21.9, 7.0 and 2.0 per 1,000 child years respectively. Unadjusted RSV admission rates were higher throughout the first 3 years of life for children with chronic conditions, born at less than 34 weeks of gestation, with a postnatal stay of more than 14 days or a 5-minute Apgar score of less than 8 (Table S2). After the first year of life, RSV admission rates were similar by season of birth and size for gestational age.

In the fully adjusted model, premature birth, presence of chronic conditions, birth between July and December and being small for gestational age were the birth characteristics independently associated with an increased risk of RSV admission (Table 2). Being born post-term was associated with a decreased risk. Of the family characteristics, having one older sibling was associated with an 80% increased risk of RSV admission (hazard ratio (HR) 1.80; 95% confidence interval (CI): 1.68­–1.93), and having two or more older siblings was associated with double the risk of RSV admission compared with children without older siblings (HR 2.18; 95% CI: 2.02–2.35). Further, maternal smoking during pregnancy and younger maternal age were associated with an increased risk of RSV admission.

**Table 2:** Crude and adjusted hazard ratios for risk of respiratory syncytial virus hospital admission in children less than 3 years old according to risk factor, birth cohort study, Scotland, 2009–2015 (n = 5,033 admissions)

Risk factor	Crude HR (95%CI)	Adjusted^a^ HR (95% CI)
**Gestational age**		
< 34 weeks	3.87 (3.42–4.38)	2.54 (2.10–3.06)
34–36 weeks	1.71 (1.53–1.92)	1.58 (1.41–1.77)
37–40 weeks	1	1
≥ 41 weeks	0.74 (0.69–0.80)	0.81 (0.75–0.87)
**Sex**		
Boys	1	1
Girls	0.84 (0.79–0.89)	0.85 (0.81–0.90)
**Chronic condition recorded before 6 months** ^b^		
No	1	1
Yes	3.40 (3.11–3.72)	2.67 (2.42–2.94)
**Postnatal stay in hospital**		
≤ 14 days	1	1
> 14 days	3.93 (3.41–4.53)	1.11 (0.90–1.37)
**Season of birth**		
January–March	1	1
April–June	0.95 (0.87–1.04)	0.94 (0.86–1.03)
July–September	1.37 (1.26–1.49)	1.37 (1.26–1.49)
October–December	1.74 (1.61–1.89)	1.77 (1.64–1.92)
**Apgar score at 5 min**		
0–7	1.75 (1.51–2.02)	1.11 (0.95–1.29)
8–10	1	1
**Birthweight by gestational age**		
Small for gestational age	1.25 (1.14–1.38)	1.14 (1.03–1.25)
Right for gestational age	1	1
Big for gestational age	0.95 (0.86–1.04)	1.02 (0.92–1.12)
**Number of siblings (parity)**		
None	1	1
1	1.67 (1.57–1.79)	1.80 (1.68–1.93)
≥ 2	2.07 (1.92–2.22)	2.18 (2.02–2.35)
**Maternal smoking during pregnancy**		
No	1	1
Yes	1.51 (1.42–1.62)	1.29 (1.20–1.39)
**Maternal age**		
< 20 years	1.10 (0.98–1.24)	1.43 (1.26–1.63)
20–29 years	1.14 (1.08–1.21)	1.24 (1.16, 1.32)
30–39 years	1	1
≥ 40 years	0.88 (0.74–1.04)	0.77 (0.66–0.92)
**SIMD quintile**		
1 Most deprived	1.18 (1.08–1.29)	0.91 (0.83–1.00)
2	1.09 (0.99–1.20)	0.91 (0.83–1.01)
3	1.02 (0.93­–1.12)	0.92 (0.83–1.02)
4	1.03 (0.94–1.14)	0.98 (0.89–1.09)
5 Least deprived	1	1

CI: confidence interval; HR: hazard ratio; SIMD: Scottish Index of Multiple Deprivation.

^a^ Adjusted for all other variables in model.

^b^ Including congenital heart disease, congenital malformations of the respiratory system, neurological disease and chronic lung disease.

The 5,033 admissions were in 169,726 children.

Delayed infant vaccination was associated with an independent and statistically significant increased risk of RSV admission (adjusted HR 1.14, 95% CI: 1.03–1.25; see Table S3). The increased risk associated with presence of older siblings was substantially attenuated for children aged over 6 months (e.g. adjusted HR for presence of one sibling 1.44; 95% CI: 1.31–1.59; Table S3) Complete case analyses were very similar to models based on multiple imputation (Table S4).

Only 6.5% of RSV admissions would be prevented if the excess risk among children with chronic conditions was eliminated (Table 3). If all children had similar risks to post-term babies, 19% of RSV admissions could be prevented. However, removing the risk posed by older siblings or due to lower maternal age would reduce the number of RSV admissions by over 30%.

**Table 3:** Population attributable fraction (as a percentage of admissions prevented) by risk factor and scenario, birth cohort study, Scotland, 2009–2015

Risk factor and scenario	Population attributable fraction (95% CI)
**Gestational age = post term**	**18.9 (13.8–23.7)**
Season of birth = April–June	25.6 (21.0–30.0)
Chronic condition = No	6.5 (5.6–7.5)
Parity = 0	34.0 (31.0–36.9)
Maternal smoking during pregnancy = no	5.9 (4.2–7.7)
Maternal age = ≥ 40 years	31.4 (18.7–42.1)
Birth weight at gestational age = right for gestational age	1.6 (0.1–3.0)
Delayed infant vaccination = no^b^	2.5 (0.5–4.5)

CI: confidence interval.

^a^ Population attributable fraction of admissions prevented if the particular risk factor was set to that for the specified risk group for all children; all other variables are kept the same.

^b^ Children aged ≥ 6 months only.

## Discussion

Half of all RSV admissions in the first 35 months of life occur during the first 6 months, and 30% of admissions after age 1 year. Although children born with chronic conditions were at significantly increased risk of RSV admission throughout the first 3 years of life, only 19% of RSV admissions occurred in these high-risk children. Children with older siblings were twice as likely to be admitted with RSV infection and we estimated that reducing this risk would reduce the number of RSV admissions by nearly one third. We also identified a 14% increased risk of RSV admission among children who had a delay in completing the infant vaccination programme.

This is the most comprehensive study of RSV admissions in the UK to date, and one of the few large-scale studies for RSV admission in the literature using laboratory data to confirm RSV infection [14,25,28]. A key strength of this study was the use of a national birth cohort including all singleton births in Scotland over 3 years with follow-up through linkage to administrative health databases. This allowed us to quantify the contribution of each risk factor through calculation of population attributable fractions. Our approach also ensured minimal selection bias, loss to follow-up, and sufficient numbers to examine risks in small subgroups of children, including children born at less than 34 weeks’ gestation. The well-established national data linkage infrastructure and universal recording of the CHI number on all healthcare interactions in Scotland enabled the examination of both clinical and family risk factors, and the association between vaccination history and risk of RSV admission. Rich administrative data resources such as this are required to measure the impact of a future RSV vaccination programme.

A limitation of using linked administrative health databases is that testing for RSV in children presenting to hospital is likely to favour inclusion of children with more severe symptoms and those in high-risk groups. This is because there is no national swabbing and testing programme for children presenting to hospital with symptoms of respiratory infections in Scotland or elsewhere in the UK. Testing practices are likely to vary both according to hospital and child characteristics, as well as time of year. It is likely that younger children, those with more severe symptoms, children born prematurely or with chronic illness are more likely to be tested. This may have underestimated the number of RSV admissions in older children. Likewise, length of stay would be longer for children who are tested for RSV if the likelihood of testing is related to increased illness severity; hence the estimates presented here may not be representative of all RSV admissions. If the probability of being swabbed and tested is higher among children with underlying health problems, this would lead to an overestimation of the hazard ratios and population attributable fractions for presence of chronic conditions and premature birth. A second limitation is that we could not define some risk factors explored in previous studies, such as early socialisation through group childcare [29], or mode of delivery [30]. Finally, we could not account for children who received prophylactic treatment with palivizumab since there is no national individual-level hospital prescribing database in Scotland (or elsewhere in the UK).

Our findings have important implications for the design of a future RSV vaccination programme and for policies to prevent RSV admissions in the pre-vaccine era. As reported in other studies [17,25,28], we identified an increased risk of RSV admission among children born prematurely and with chronic conditions, and RSV admission rates in these children remained higher than among low-risk children across the first 3 years of life. Children in these high-risk groups accounted for a disproportionate number of bed days. Therefore, any future RSV vaccination programme needs to ensure that these children are protected until at least 3 years of age. Maternal vaccination is an attractive strategy since it would protect the youngest infants, however, it would require careful timing during pregnancy to ensure premature babies are protected.

We identified presence of older siblings as an important risk factor for RSV admission. Older siblings are likely to spend substantial amounts of time in closed settings outside the home (nurseries and schools) where infection risk is high [31]. A household study in Kenya identified older siblings (the vast majority of whom were attending school) as the source of nearly three quarters of infant infections [13]. Children are also more likely than adults to spread infection to infants due to frequent hand-to-mouth contact, and lack of hygienic practices (such as frequent handwashing and sneezing into tissues rather than hands).

Eliminating the risk posed by older siblings could reduce the number of RSV admissions by a third across the first 3 years of life. Vaccinating older siblings could therefore be an important strategy in the UK and other high-income countries in order to prevent RSV admissions among the youngest children [14]. As Poletti et al. [32] demonstrate in a low-income country setting, there are different strategies through which protection from infection risk posed by older siblings could be incorporated into an RSV vaccination programme. For example, it could be achieved by a school-based vaccination programme. Such a programme would lead to a reduction in population-level transmission as well as directly protect young siblings of school-aged children from infection. Alternatively, sibling vaccination could complement a newly introduced routine infant vaccination programme for the first few years after introduction; however, the effect would wane with the number of years since introduction. Ultimately, the optimal design of an RSV vaccination programme which effectively prevents infections and hospital admissions among infants and young children will depend on the availability, effectiveness, safety, likely uptake and cost of vaccines for different age groups. However, our results indicate that vaccination of older children should be considered as a potential scenario in future cost-effectiveness models for RSV vaccination programmes in a UK context. Further studies are required to determine the risk of RSV admission according to the age of older siblings.

Our results suggest other measures to reduce the spread from older siblings are also likely to reduce RSV admissions. A systematic review has identified evidence from several clinical trials that handwashing is effective in reducing transmission of respiratory viruses from younger children in particular [33]. Interventions to limit transmission from siblings should focus on infants born between July and December, who had the highest risk of RSV admission in our study.

The risk of RSV admission in this study increased with decreasing maternal age. An increased risk of RSV admission with younger maternal age has also been reported in other studies [28,34]. This is likely explained by the strong association between young maternal age and low socioeconomic status. Further studies with more detailed data on living standards are required to explore which aspects of socioeconomic status, such as poor housing quality [35,36], explain the observed increased risk of RSV admission in children with younger mothers. In addition, low socioeconomic status (as indicated by young maternal age) is associated with premature birth and intrauterine growth restriction, which in themselves are risk factors for RSV admission, as we have demonstrated. Thus, the total contribution of young maternal age or maternal smoking to the risk of RSV admission may be underestimated in this study. Future work should also examine the causal pathways through which low socioeconomic status affects the risk of RSV admission.

We observed a small but significant increase in RSV admissions associated with delayed infant vaccination. Our study is the first to examine the role of timely infant vaccination and the risk of RSV admission in a high-income country setting [15]. None of the current infant vaccines are expected to provide direct protection from RSV infection (only secondary protection through prevention of bacterial co-infection). Delayed infant vaccination could indicate a lack of access to preventive health services. However, children whose infant vaccinations are delayed or incomplete are more likely to be from poor socioeconomic backgrounds or have long-term chronic illness [37-39], which may explain the association observed in this study.

Our study highlights that any future RSV vaccination programme will need to protect children throughout the early life course, and in particular children with chronic conditions, who remained at increased risk throughout the first 3 years of life. Further, protecting young children from infection risk posed by older siblings, including through vaccination of older children, could have a substantial impact on reducing RSV admissions.
