# Erratum for Euro Surveill. 2019;24(1)

**DOI:** 10.2807/1560-7917.ES.2018.24.3.1917011

**Published:** 2019-01-17

**Authors:** Eurosurveillance editorial team

**Affiliations:** 1 European Centre for Disease Prevention and Control (ECDC), Stockholm, Sweden

In the article ‘The contribution of child, family and health service factors to respiratory syncytial virus (RSV) hospital admissions in the first 3 years of life: birth cohort study in Scotland, 2009 to 2015’, published on 3 January 2019, a general note under Table 3 was erroneously labelled as note a. This was corrected and the remaining note was reordered on 10 January 2019.

